# Re-Education of the Tabetic

**Published:** 1908-03-28

**Authors:** G. S. Parkinson


### The General Practitioner's Column.

[Contributions to this Column are invited, and if accepted will be paid for.]

### RE-EDUCATION OF THE TABETIC.

BY G. S. PARKINSON, M.R.C.S., L.R.C.P.

It it pleasing to think that the case of the tabetic is not so hopeless as it was at one time considered; I speak now with regard to his re-education in the art of walking. While spending some weeks in Ghent this year, I was permitted through the kindness of the hospital authorities, to witness the treatment of numerous cases of locomotor ataxia. The hospital, the greater part of which is new, has a most perfect installation for electro-therapy and mechano-therapy. No expense seems to have been spared in providing the necessary apparatus.

A record is kept of the visits of the patients, and in the electro-therapy department the strength of current employed at each visit. In the mechano-therapy department a most elaborate record is kept. All exercises are divided into a series of three. These are again sub-divided into three exercises in each:—

1. (1) Arms, (2) back, (3) legs.
2. (1) Back, (2) legs, (3) arms.
3. (1) Legs, (2) arms, (3) back.

Exercises are given to arms, back, and legs in an alternate manner, so that the patient does not get tired. Each exercise lasts for three minutes, and between each two minutes' rest is given.

The vibrator appears to play an important part in the treatment of these cases. The patient, sitting down, puts both legs on a leather cushion supported on a metal stem; he then grasps two handles in his hands. When this machine (which can be started and stopped by means of a small lever) is set in motion the whole machine vibrates. This is said to have a very stimulating effect on the muscles.

He then passes on to the next machine for passive flexion and extension of the wrist. Resting his hands on a flat metal bar, another similar bar comes in contact with the back of the hands, and thus gently secures them. The machine works with a pendulum-like movement, giving passive flexion and extension to the wrist joint. A similar course of passive flexion and extension is given to the ankle and knee joints. To those who suffer from what is commonly known as "pins and needles" in the foot, massage is given to the soles of the feet. The patient sits down, and places both feet on a drum, which is ribbed, and covered with a large piece of chamois leather, which is thrown loosely over it; this drum revolves at a moderate rate. This is given for one or two minutes. The time taken over these exercises is marked by a three-minute sand-glass.

Such a course of treatment lasts eight to ten days, and it is well to note that nothing but passive movement has been so far allowed. After this period both active and passive movements are commenced. The patient is now given pronation and supination of the arm; the arm is pronated fifteen times, and again is supinated fifteen times; the resistance can be graduated.

Another exercise is adduction and abduction of the arms. Sitting down, the patient takes hold of two wooden bars, about three feet long; he then abducts his arms, pressing these wooden bars back against resistance, at the same time taking a deep inspiration. This constitutes active abduction. The wooden bars then return to rest by means of weights, causing passive adduction. The machine can be reversed, so that we get passive abduction and active adduction, which causes in turn an active expiratory act.

As improvement takes place, more exercises are given to the lower extremities, among the best of which is the bicycle exercise, in which the patient sits down and pedals, giving movement to ankle, knee, and hip joints. Adduction, abduction, and circumduction are given to both shoulder and hip joints.

Added to this, the patient has a course of electro-therapy, which is given on alternate days, with the exercises above described. It consists of electric baths to arms and legs, a current of from 10 to 15 milliamperes being passed. The patient gets ten minutes of these baths, and the current is carefully measured by an amperemeter. He is also given galvanic current to arms, legs, and spine, a negative current being often used for the arms, while a positive current is used for the legs; but this rule is not always followed. The ringing of an electric bell indicates that the patient has had his ten minutes' treatment. At the end of one month of this treatment, ten to fifteen days complete rest is given, during which time the patient does not come near the institution.

To some cases hot and cold shower douches are given, and there is a most admirable installation of baths of every description.

The results gained by this method of treatment are, to say the least of it, most encouraging. I was very much interested in the case of a woman, which demonstrated the importance of this system of treatment. She came to the Institution displaying such ataxic symptoms that progression was almost impossible; at the end of forty-three visits to the mechano-therapy department, with a similar number to the electro-therapy department, she was able to walk down the room without assistance—a feat which was quite impossible before.

